# An Efficient and Smooth Methadone-to-Buprenorphine Transition Protocol Utilizing a Transdermal Fentanyl Bridge and a Pharmacokinetic Inducer: The Stanciu Method

**DOI:** 10.7759/cureus.8310

**Published:** 2020-05-27

**Authors:** Cornel N Stanciu, Stephen Gibson, Nikhil Teja, Christopher J Healey

**Affiliations:** 1 Psychiatry, Geisel School of Medicine, Concord, USA; 2 Pharmacy, New Hampshire Hospital, Concord, USA; 3 Psychiatry, Dartmouth-Hitchcock Medical Center, Lebanon, USA; 4 Substance Abuse Counseling, New Hampshire Hospital, Concord, USA

**Keywords:** moud, buprenorphine, methadone, methadone to buprenorphine, mat, medication assisted therapy, opioid use disorder, pain management

## Abstract

The traditional method of transitioning from methadone to buprenorphine requires a gradual dose reduction to a low dose of 30 mg daily, followed by cessation, and addressing withdrawal symptoms prior to the initiation of buprenorphine. This process can be time-consuming and is also associated with tremendous patient suffering and adverse outcomes. In recent years, several protocols have emerged based on the notion of blunting the shift from full receptor activation to partial receptor activation via an intermediate “bridge”. This typically is required for the time period needed for the acting full agonist, methadone, to undergo biotransformation and clearance. In this report, we present an inadvertent case where transdermal fentanyl as a transitional bridge was utilized along with an inducer of methadone’s metabolism to speed up the course, and urine acidification to enhance clearance. Our patient was transitioned from moderate-dose methadone, without encountering any withdrawal symptoms in the process, in three days. This method presents yet another option for select candidates, and it allows physicians to individualize methadone-to-buprenorphine transitions.

## Introduction

Methadone and buprenorphine are widely used in pain management as well as treatment programs for patients with opioid use disorder (OUD). The choice of agent involves a careful biopsychosocial assessment of the patients, taking into account their history, medical comorbidities and substance use, geographical availability of resources, and past treatments among others. At times, transitioning from methadone to buprenorphine may be necessary. This can be a very torturous process as methadone has a long half-life and extremely unpredictable pharmacokinetics, while buprenorphine is only a partial agonist, however, with a very high affinity for the same involved opioid receptors. The traditional protocol involves a gradual dose reduction to 30 mg daily of methadone, followed by cessation for a period of up to 96 hours during which patients can become extremely uncomfortable as they transition through periods of withdrawal, prior to finally being able to induce on buprenorphine [1]. Administration of buprenorphine too early can suddenly displace methadone from the receptors and precipitate withdrawal symptoms through a sudden drop from full to partial receptor activation. The overall transition can not only derail sobriety in the context of OUD but is extremely uncomfortable for the patient.

In recent years, several protocols have emerged, each having been crafted for specific treatment settings, based on involved cost and availability of formulations [2]. They all attempt to balance patient comfort with the time required to perform the buprenorphine induction. Their shared characteristic is the notion of “bridging”, first pioneered by Hämmig et al. [3]. This entails a very slow loading of the high-affinity buprenorphine on to the opioid receptors without causing a drastic displacement of the full agonist, which is slowly eliminated by biotransformation and clearance, leading to a withdrawal-free transition. The duration of this process is 4.5 half-lives of the full agonist used before induction. One of the protocols developed by Azar et al. uses transdermal fentanyl, a highly potent full agonist, as the bridge [4]. In his method, methadone is replaced with an equivalent dose of fentanyl for five days. As the patch is removed, the buprenorphine induction begins. Fentanyl has a slightly weaker, if not very similar, binding affinity for the mu receptor as buprenorphine; yet it provides the same full activation as methadone [5]. As methadone dissipates, fentanyl occupies the receptors, and when buprenorphine is eventually introduced, it will not cause a large displacement at the receptor, but rather it will be more competitive and gradual. The transdermal system builds up fentanyl in the skin, resulting in a half-life of 17-27 hours and requiring up to five days to fully eliminate. This protocol can take 8-13 days for full induction.

In this report, we review methadone’s profile and introduce a modified version of the fentanyl patch protocol that incorporates a pharmacokinetic inducer of methadone to provide a faster and smoother transition to buprenorphine: the Stanciu method.

Methadone pharmacokinetics

Methadone is lipophilic, and on ingestion, it is rapidly absorbed with plasma detection occurring within 30 minutes, with peak levels attained within 1-7.5 hours. With chronic daily ingestion, 80% of it passes into the bloodstream and the remainder undergoes metabolism in the liver and gastrointestinal tract (GI) [6]. From vasculature, it is extensively distributed at 1-8 L/kg throughout tissues such as the brain, intestines, kidney, liver, muscles, and lungs, thereby building a storage reserve [7]. When chronic use is stopped, there is a gradual and slow process of redistributing from these tissues intravascularly. Although tissue storage is superior to its existence in plasma, the ratio can be quite variable among patients. In plasma, methadone is highly bound to proteins and its mean free fraction is 13% with a four-fold individual variability [6].

Metabolism occurs in the liver and to a lesser extent in GI, with excretion occurring renally and fecally. Its half-life is ~22 hours and there is tremendous individual variability of 8-150 hours [8]. The cytochrome P450 isoenzymes thought to be involved are 3A4, 2B6, and, to a lesser extent, 2C19, 2D6, and 2C9. Although 3A4 was the primary enzyme hypothesized to be responsible for biotransformation historically, recent evidence points to CYP2B6 [9]. Some evidence suggests that pharmacokinetics are completely independent of CYP3A [10].

The bodily clearance is highly variable (0.02-2L/min) and, given the redistribution from storage, plasma concentration decreases bi-exponentially [6]. As a basic compound, methadone’s excretion is dependent on urinary pH. When pH is >6, renal excretion is responsible for 4% of the excreted drug, while at pH of <6, 30% is excreted by the kidneys.

## Case presentation

The patient was a 37-year-old Caucasian female suffering from depression, chronic low back pain, and OUD in sustained remission, who was admitted to the inpatient psychiatric unit for suicidal ideations. She endorsed four months of despondency surrounding the anniversary of her infant’s death. This triggered recreational illicit use, with admission urine toxicology revealing methamphetamines, benzodiazepines, and methadone. The outpatient regimen consisted of methadone 40 mg twice daily and gabapentin 600 mg four times daily.

Our addiction service was consulted for advice on how to proceed. Her substance use history was discussed, and it emerged that she had been introduced to smoking cocaine at an early age by her mother who had suffered from addiction herself. Although she had not found it enjoyable at the time, she had developed an affinity to use in social gatherings in her late teens. A back injury following a car accident at age 20 had led her to take prescribed short-acting opioids, which she had quickly begun to misuse. She had been discharged from the clinic and started obtaining these drugs illicitly with a quick transition to intravenous heroin use. Following several overdoses and legal repercussions, her father had prompted her to seek treatment at a methadone clinic, and she had been able to maintain remission for 10 years. She had found great analgesic relief with a 160-mg daily dose, allowing her to be functional. Due to a relocation and subsequent lack of access to a methadone clinic, she had transitioned to a local pain management clinic for methadone three years prior to the current admission.

Her recent relapse involved methamphetamines and benzodiazepines; however, she disclosed having misused methadone for euphoria, sometimes taking more than her daily prescribed dose. The pain clinic had developed concerns over her hepatitis C (diagnosed several years ago) and potential addition to cardiotoxic psychotropics for mood and had, prior to this admission, begun to reduce her daily dose while considering buprenorphine.

On admission, the laboratory tests revealed aspartate aminotransferase (AST) and alanine aminotransferase (ALT) of 120 and 107 IU/L respectively, with normal renal function. Her prior methadone usage had been 40 mg twice daily (BID), and, on admission, this was reduced to 30 mg twice daily with a plan to transition to buprenorphine. She was also started on duloxetine 30 mg daily for mood disorder. In the first few inpatient days, she relied heavily on ibuprofen, and attempts to manage low back pain were ineffective despite concurrent physical therapy. She experienced mild opioid withdrawal four days post-admission, requiring three clonidine 0.1-mg doses. On the morning of the fourth inpatient day, the Clinical Opiate Withdrawal Scale (COWS) score was found to be 7 when measured before administering the morning methadone dose; this was driven primarily by secondary issues of subjective anxiety, pain, and restlessness. After a discussion regarding various transitioning protocols, methadone was stopped, and a 25-mcg transdermal fentanyl patch was applied to manage the worsening back pain at 8:00 PM. That morning, she was started on carbamazepine 200 mg BID. The COWS score was found to be 2 after the fentanyl patch was applied and the pain was adequately controlled; she did not require any further nonsteroidal anti-inflammatory drug (NSAIDs).

On day one of the transition protocol, she was free of any discomfort, and COWS was 0 with no clonidine required. She was advised to avoid citrus and dairy products and consume cranberry juice if possible. Day two of the protocol was also uneventful; however, on day 3, the patch came off at 11:00 AM when she took a shower. The patient made an informed request to attempt a quicker-than-anticipated induction to buprenorphine. She received 1-mg buprenorphine at 1:00 PM and then again at 2:00 PM. She then received three more doses of 2 mg at two- hour intervals. COWS score remained 0, and she described no symptoms of discomfort and needed no clonidine. Carbamazepine was stopped after the evening dose and, on day four, she tolerated 8 mg BID of buprenorphine while COWS remained 0, and the pain was controlled with split dosing.

**Figure 1:**
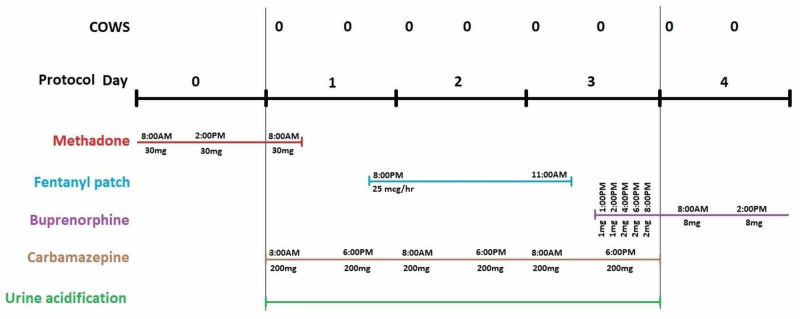
Descriptive visual of the protocol utilized in our case COWS: Clinical Opiate Withdrawal Scale

It is important to note that this is a retrospective report as the initial intent did not involve the adoption of this particular protocol (Figure 1). The treatment was administered for the sole benefit of the patient, whose consent was obtained for reporting this case in medical literature, and not to generate generalizable knowledge.

## Discussion

We presented a symptom-free, three-day transition from moderate-dose methadone to buprenorphine using a transdermal fentanyl patch “bridge” and an inducer of methadone metabolism. Although protocols for transferring patients from low-dose methadone (<50 mg) are uncomplicated, those for moderate and high-doses are complex and limited in number. The risk of precipitated withdrawal is high with doses upwards of 50 mg daily, something we did not encounter here. Additionally, female gender and a short time to induction are also risk factors predisposing to withdrawal [11].

Evolving from the fentanyl patch protocol developed by Azar et al. to transition a male patient from 30-mg daily methadone, we incorporated pharmacokinetic principles to shorten the duration of the bridge. According to current literature, CYP isoform 2B6 is primarily responsible for methadone’s metabolism, while 3A4 is also involved to some, yet now unclear, degree. Carbamazepine administration certainly leads to withdrawal in methadone-dependent patients and plasma methadone levels exhibit a 60% drop seven days post-carbamazepine initiation [12,13]. Carbamazepine was demonstrated to cause a 14-fold activation of 2B6, and an eight-fold activation of 3A4 [14]. However, enzymatic induction occurs in three days for CYP2B6 while 14 days are required for 3A4, which was consistent with our case [15]. In addition to induction of metabolism, our patient was also advised to avoid consumption of citrus and dairy products and to drink ample cranberry juice. The underlying principle is that urine acidification assists in renal methadone elimination [16].

Our method has several limitations. Firstly, high doses of methadone or underestimating the equivalent fentanyl conversion may lead to withdrawal symptoms, while overestimating may result in an overdose. Using morphine equivalents, the highest dose of methadone that could be converted to fentanyl patch intermediate is 260 mg [17]. However, our method’s conversion ceiling is limited by buprenorphine’s profile. Being a partial receptor activator, it requires higher mu occupancy than the full activators for equivalent effects. Some PET studies have shown that for patients on maintenance methadone doses (standard range being 60-120 mg daily), a ~30% mu occupancy is required [18]. And to achieve the same effect with buprenorphine, an 80-98% occupancy is required, corresponding to doses of 16-32 mg daily [18,19]. Hence, ~120 mg methadone may perhaps be our method’s conversion ceiling, and in our case, we were cautious in underestimating the methadone to transdermal fentanyl conversion to accommodate for potential cross-tolerance given methadone’s long half-life and unpredictable kinetics [20]. Our method may not be applicable to outpatient settings where there is unsupervised access to this highly potent opioid, and if inadvertent overdoses occur, it would be difficult to intervene. This method may also be limited by drug-to-drug interactions with the concurrent pharmacological regimen, although the short duration of carbamazepine does not impact CYP3A4. Lastly, not all countries allow the use of an opioid during withdrawal. Here, the fentanyl patch was primarily used to manage recalcitrant pain in the absence of methadone; however, it ended up secondarily providing our desired effect.

**Figure 2:**
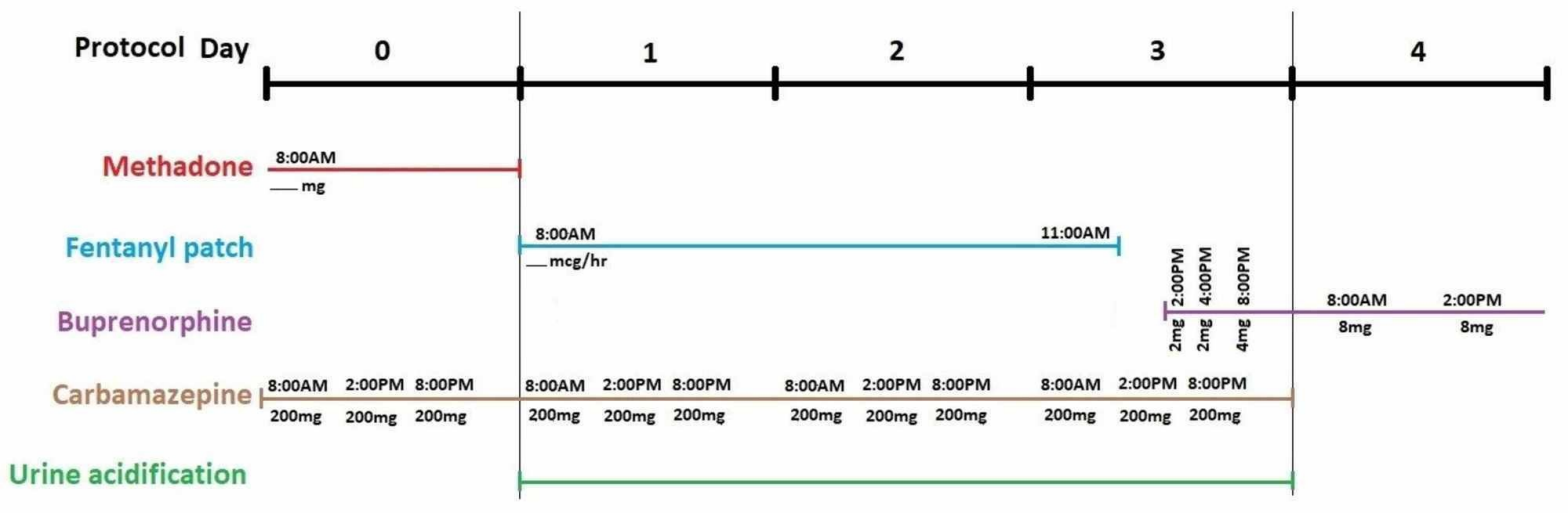
Proposed protocol for transitioning from methadone to buprenorphine using a fentanyl transdermal bridge and CYP inducer along with urine acidification for enhanced methadone clearance

Future studies should involve piloting this method as a structured trial involving subjects to allow for an evaluation of safety and efficacy. One modification should involve dosing carbamazepine three times daily, congruent with enzyme induction studies, and starting it a day prior to methadone cessation to maximally induce the involved CYP enzyme [15]. Secondly, given fentanyl’s receptor affinity, a more aggressive buprenorphine induction could be attempted as generating a 1-mg dose via strip splitting is clinically challenging. Finally, attempts should be made to determine the methadone dose ceiling that can be directly converted with this method. This proposed method with the discussed adjustments is depicted in Figure 2. One aspect to monitor is whether abrupt discontinuation of methadone, an NMDA antagonist, from higher doses leads to clinical glutaminergic rebound, in which case the addition of a short course of gabapentin can be considered.

## Conclusions

Currently, several protocols to support the transition from low-dose methadone to buprenorphine exist. The method described here represents yet another technique that can achieve the transition from moderate and higher-dose methadone in a symptom-free manner over three days. Physicians should individualize treatment and select protocols based on the specific settings, availability of products, cost, time constraints, as well as patient-driven informed choice.
